# Comparative Epigenomic Profiling of the DNA Methylome in Mouse and Zebrafish Uncovers High Interspecies Divergence

**DOI:** 10.3389/fgene.2016.00110

**Published:** 2016-06-17

**Authors:** Chi Zhang, Yujin Hoshida, Kirsten C. Sadler

**Affiliations:** ^1^Department of Medicine/Division of Liver Diseases, Icahn School of Medicine at Mount Sinai, New YorkNY, USA; ^2^Department of Developmental and Regenerative Biology, Icahn School of Medicine at Mount Sinai, New YorkNY, USA; ^3^Liver Cancer Program/Tisch Cancer Institute, Icahn School of Medicine at Mount Sinai, New YorkNY, USA; ^4^Program in Biology, New York University Abu DhabiAbu Dhabi, UAE

**Keywords:** DNA methylation, comparative epigenomics, mouse, zebrafish, liver, brain

## Abstract

The DNA methylation landscape is dynamically patterned during development and distinct methylation patterns distinguish healthy from diseased cells. However, whether tissue-specific methylation patterns are conserved across species is not known. We used comparative methylome analysis of base-resolution DNA methylation profiles from the liver and brain of mouse and zebrafish generated by reduced representation bisulfite sequencing to identify the conserved and divergent aspects of the methylome in these commonly used vertebrate model organisms. On average, 24% of CpGs are methylated in mouse livers and the pattern of methylation was highly concordant among four male mice from two different strains. The same level of methylation (24.2%) was identified in mouse brain. In striking contrast, zebrafish had 63 and 70% of CpG methylation in the liver and brain, respectively. This is attributed, in part, to the higher percentage of the zebrafish genome occupied by transposable elements (52% vs. 45% in mice). Thus, the species identity was more significant in determining methylome patterning than was the similarity in organ function. Conserved features of the methylome across tissues and species was the exclusion of methylation from promoters and from CpG islands near transcription start sites, and the clustering of methylated CpGs in gene bodies and intragenic regions. These data suggest that DNA methylation reflects species-specific genome structure, and supports the notion that DNA methylation in non-promoter regions may contribute to genome evolution.

## Introduction

Cytosine methylation is a major epigenetic mark in many species, as it serves to significantly alter the accessibility of CpG sites across the genome (Deaton and Bird, 2011). The methylome changes dramatically during development (Singer et al., 2014; Zhao et al., 2014), and there are marked differences in pattern of methylation across different cell types and in many pathologies, most notably, cancer. Indeed, many studies using model organisms demonstrate a required role for DNA methylation in regulating vertebrate development (Bird, 2002; Messerschmidt et al., 2014) and in causing cancer (Eden et al., 2003; Gaudet et al., 2003; Howard et al., 2008; Mudbhary et al., 2014). The canonical functions for DNA methylation are to promote genomic imprinting (Li et al., 1993), X-chromosome inactivation (Boumil and Lee, 2001), preservation of chromosome stability (Smith and Crocitto, 1999) and transposon repression (Slotkin and Martienssen, 2007; Zemach et al., 2010); these functions are largely conserved across taxa. However, not all species methylate their genomes, and those that do exhibit a wide range of DNA methylation levels and patterns (Lee et al., 2010; Zemach et al., 2010). Even when the same region of the brain is compared between closely related species (humans and chimpanzees), marked differences in methylation levels and the pattern of methylation have been reported (Zeng et al., 2012). Whether the species-specific methylome difference reflects functional differences played by DNA methylation in different organisms or, instead, whether they mirror, and perhaps contribute to, genome evolution remains an open and important question.

Many studies on DNA methylation are based on the hypothesis that methylation serves to repress gene expression (McGhee and Ginder, 1979). There are, however, many exceptions to this pattern, and, indeed, it is now widely accepted that differences in promoter methylation play a regulatory role in only a few well publicized cases. In contrast, overwhelming evidence shows that, across cell types and species, 1000s of silenced genes have entirely unmethylated promoters and, conversely, many highly expressed genes have high levels of promoter methylation (Bestor et al., 2015). While CpG islands (CpGi) exert regulatory function on gene expression, their protection from DNA methylation is a conserved feature of CpGis (Long et al., 2016). However, the picture is becoming more complex, as recent work has shown that enhancer methylation is correlated with gene expression in developing zebrafish and other vertebrates (Lee et al., 2015; Bogdanović et al., 2016) and other studies show that moderately expressed genes do not have a strong correlation with methylation differences at upstream regulatory regions, but instead these genes are highly methylated throughout the gene body (Elliott et al., 2015). Thus, the understanding of how DNA methylation impacts gene expression is evolving.

Comparative methylome analysis across taxa has uncovered widely divergent methylation patterns across species (Feng et al., 2010; Lee et al., 2010; Zemach et al., 2010), supporting the conclusion that methylation cannot be a universal mechanism of repressing gene expression, or, perhaps, that DNA methylation may serve different functions across the branches of the phylogenetic tree. In contrast, the high level of methylation of transposable elements (TEs) is a feature of DNA methylation that is conserved from plants to animals (Zemach et al., 2010). This is proposed to be a central mechanism to repress the potentially catastrophic activation of these endogenous parasites (Yoder et al., 1997). Comparative studies further supported the hypothesis that DNA methylation function is nuanced and variable. Moreover, the stark differences in DNA methylation between vertebrates and invertebrates limit studies on the functional consequences of methylome repatterning to vertebrate models such as mouse and zebrafish.

We reasoned that if DNA methylation is a central mechanism of regulating gene expression, then this should be reflected in conserved methylation patterns of genes that have similar expression patterns across species. The liver serves a fundamental role in metabolic homeostasis in all vertebrates, and this is reflected in a shared pattern of gene expression from humans to fish (Lam et al., 2006; Mudbhary et al., 2014). We selected this organ for comparative methylome analysis between mouse and zebrafish because there are striking similarities in liver cell composition and function (Goessling and Sadler, 2015). Studies in zebrafish also show that accurate DNA methylation is essential for liver development and regeneration (Sadler et al., 2007; Jacob et al., 2015) and that loss of methylation causes liver cancer (Mirbahai et al., 2011).

Multiple approaches have been developed and used to characterize genome-wide DNA methylation (Reid, 2010). Of these, reduced representation bisulfite sequencing (RRBS) is a cost-efficient method to survey CpG methylation genome wide by sampling regions of the genome that are CpG rich for bisulfite sequencing (Gu et al., 2011). A drawback to this approach is that it mainly covers CpG islands at the expense of other genomic regions, In order to represent more CpG sites and increase the coverage of all genomic regions interrogated, a recently published enhanced RRBS (ERRBS; Akalin et al., 2012; Garrett-Bakelman et al., 2015) allows greater distribution across the genome of the mapped reads from bisulfite converted genomic DNA. Hepatic function and the population of hepatic cells is similar between zebrafish and mammals (Goessling and Sadler, 2015) and we asked whether these similarities would be reflected by conserved features of the methylome. We used ERRBS to generate single-nucleotide resolution DNA methylation map of the mouse liver and compared this to a previously described methylome analysis of zebrafish liver generated by RRBS. We report marked interspecies differences in methylation patterns, and by comparing the methylation landscape between liver and brain of mouse and zebrafish, we found that the difference between species is much more significant than differences between tissues of the same species. Our findings suggest that genome structure is the primarily determinant of methylome patterning.

## Materials and Methods

### Sample Preparation

The four mouse samples (Mixed-1, Mixed-2, B6-1, and B6-2) that were used for bisulfite sequencing are all males and are from two different genetic backgrounds: B6-1 and B6-2 are C57BL/6 mice, whereas the mixed mice are on an undefined background including C57BL/6J and other strains. Total livers were isolated from them between 6 and 8 weeks old and frozen at -80°C for DNA isolation. Genomic DNA was extracted from each sample using the Qiagen DNA isolation kit according to the manufacturer’s instruction. RNA was purified using Qiagen RNAeasy columns. The animal work was approved by Institutional Animal Care and Use Committee (IACUC) at the Brigham and Women’s Hospital (mixed samples) and the Memorial Sloan Kettering Cancer Center (C57BL/6 samples).

### Enhanced Reduced Representation Bisulfite Sequencing (ERRBS) Library Preparation and Sequencing

Bisulfite-converted DNA sequencing libraries were generated for each of the studied samples, which measure both 5hmC and 5mC in methylated fraction and 5fC, 5caC, and C in unmethylated fraction. In brief, 50 ng of high quality genomic DNA in 50 μl of DNase-free water was used as starting material. The whole library preparation includes enzyme digestion of genomic DNA which enriched CpG rich regions, Phenol:chloroform clean up, End-repair, A-tailing, adapter ligation, size selection, bisulfite conversion, enrichment PCR, and quality control. Experimental details can be referred to the published ERRBS protocol (Smith et al., 2009; Garrett-Bakelman et al., 2015). These amplified libraries were sequenced on the Hiseq2000 platform for 50 cycles single end read runs at the Epigenomics Core facility in the Department of Medicine, Weill Cornell Medical College (New York, NY, USA). Image capture, analysis and base calling were performed using Illumina’s CASAVA 1.8. Data is available in GEO, accession numbers are GSM2136660, GSM2136661, GSM2136662, and GSM2136663.

### RNA Sequencing Library Preparation and Sequencing

For each of the mouse samples, Mixed-1, Mixed-2 and an additional mixed background age-matched mouse from the Brigham and Women’s facility (Mixed-3), RNAseq libraries were prepared according to Illumina’s TruSeq RNA sample preparation version2 protocol. The 3 samples were sequenced using Illumina’s HiSeq 2500 platform (100bp paired-end sequencing) generating ∼64 million reads, ∼60 million reads and ∼53 million reads for samples Mixed-1, Mixed-2, and Mixed-3, respectively.

The sequencing quality was assessed using FASTQC (Andrew, 2010) and the reads were quality trimmed using Trimmomatic (Bolger et al., 2014; for low *Q*-scores, adapter contamination and systematic sequencing errors). After quality trimming, only the fragments that retained the forward and reverse reads were kept, any reads with a length less than 36 bp after quality trimming were discarded. For all three samples, the quality trimming step resulted in a ∼3% data loss.

The reads were then aligned to the Mus Musculus GRCm38.p4 reference genome assembly using TopHat (Kim et al., 2013) version 2.1.0. Overall alignment rates were 97% for Mixed-1, 98% for Mixed-2, and 97% for Mixed-3. The resulting BAM alignment files were then passed through Cufflinks (Trapnell et al., 2012) version 2.2.1 in order to calculate gene level FPKM values.

### Read Alignment and Methylation Calls

Enhanced reduced representation bisulfite sequencing reads were mapped on the mouse genome (mm10) with the use of the BSMAP mapping tool (Xi and Li, 2009). The percentages of DNA methylation levels based on bisulfite conversation yield were computed at the single-nucleotide scale. We simulated a range of genome coverage from 5 to 20 and applied statistical analysis of differential methylation which demonstrated that only positions represented by at least 10× coverage (i.e., CpG_10_) yielded a dataset that had sufficient statistical power for differential methylation calls and allowed for a maximal number of CpG sites to be analyzed, similar to analysis carried out by others (Akalin et al., 2012). We were able to establish the DNA methylation state for 7,492,706 Cytosine positions, including 1,519,053 Cytosine positions from CpG dinucleotides at CpG_10_ across all four mouse liver samples. DNA methylation levels of the different genomic elements were computed as a mean of percentages of DNA methylation levels for all CpG dinucleotides, for which data were available in these regions.

### Methylation Comparison and Statistical Analysis

Percent methylation values for CpG dinucleotides were calculated by dividing the number of methylated Cs by sequencing depth on that base. For representation as a histogram, percentages were grouped within a 10-percentile range from 0 to 100% methylation. The methylation score for each of CpG dinucleotides that were covered by at least 10 reads in all samples (Mixed-1, Mixed-2, B6-1, and B6-2) was determined and then we used Pearson correlation distance and Ward’s agglomeration method to determine how similar methylation levels were for each CpG across these samples. Hierarchical clustering of the four samples was performed using the hclust function in R-3.2.1^1^. Locally weighted polynomial regression (Lowess) was performed in methylation status scatterplots between each two samples to check their relationships. RRBS datasets for zebrafish brain (GSE59916), liver (GSM1456413; Chatterjee et al., 2014), and mouse brain (GSM1069659) were obtained from Gene Expression Omnibus (GEO). Fisher’s exact test was adopted for testing the methylation pattern difference between mouse and zebrafish. The mouse in the brain study is the C57BL/6 strain, brain tissue was collected at 11 weeks. The zebrafish liver methylome was a pool of five male and five female liver harvested from adult fish and the zebrafish brain methylome was from a pool of two males and two females (Chatterjee et al., 2014).

To annotate the genomic feature associate with each CpG_10_ site, both gene information (promoter, exon, intron, and intergenic) and the list of CpG islands were retrieved from UCSC table browser^2^. Specific loci were visualized using the epigenome browser^3^ (Zhou et al., 2014). CpG shores were defined as 2,000 bp flanking regions on both upstream and downstream of given CpG islands (Irizarry et al., 2009). Regions >2 kb away from CpG islands are defined as “open sea.” If a CpG shore overlapped with another island, the shores were trucked. If multiple shores were overlapping, they were merged into a single shore. Based on refseq annotation, CpG dinucleotides were classified into promoter, intron, exon, and intergenic regions. The statistical test used for the distribution of different genetic elements was Fisher’s exact test. TSS regions were defined as the 2,000 bp window centered on the transcript start site (TSS) of genes. In practice, the length of TSS regions is not equal. For global viewing, we calculated the average methylation status (mCpG/CpG) and CpG density within each subset of TSS regions and divided into 40 bins equally in the TSS plot.

### Integration of DNA Methylation and Gene Expression

Each CpG_10_ site was categorized by its methylation status as hypomethylated (<20% of reads are methylated) or hypermethylated (>80% of reads were methylated). We displayed the relative expression of genes with methylated and unmethylated CpGs overlapping with promoter regions and gene bodies in according to two common definitions: 5 kb upstream of the TSS and the ±2 kb of the TSS which includes CpGs in gene bodies. Within each region, all methylated and unmethylated CpG were counted to generate methylated and unmethylated gene lists. Then, genes were ranked based on the number of CpG sites. Gene expression was represented by FPKM values and FPKM values were log transformed to display in the heat map.

## Results

### Mouse Liver Methylome Mapped by ERRBS

Many studies in mice and humans have described widespread methylome differences in the same organ under different physiological and pathological states (Bird, 2002; Spiers et al., 2015; Zhang et al., 2015). To determine whether differences between background strains or housing conditions altered the hepatic methylome, we compared ERRBS datasets from two male mice on a mixed background (mixed-1, mixed-2) and two males on a pure Black-6 (B6-1, B62) background. Mice from different strains were housed in vivariums at different institutions. As expected, ERRBS enriched for CpG sites in the mouse liver methylome (**Table 1**). ERRBS does not distinguish between 5mC and 5hmC, and thus we are unable to differentiate the potential differential contribution of these two modifications. However, given that previous studies found a very low level of 5hmC in liver (0.03–0.06% of dG) compared to the central nervous system and the spinal cord (0.3–0.7%) in mouse (Globisch et al., 2010), we reasoned that 5hmC only contributed a small proportion of all of our methylated C calls.

**Table 1:** The liver methylome is highly consistent across mouse strains.

	Mixed_1	Mixed_2	B6_1	B6_2
Number of reads	36,624,797	32,664,237	50,483,884	49,082,208
Mapping efficiency	68.3%	67.4%	67.6%	67.7%
Conversion rate	99.86%	99.89%	99.88%	99.89%
Total # of Cytosine sequenced	336,286,562	303,362,122	473,186,484	450,385,315
Total # of CpG sequenced	76,066,210	70,835,055	104,716,169	98,093,922
# of CpGs covered >10×	1,372,559 (1.80%)	1,329,761 (1.88%)	1,557,387 (1.49%)	1,528,130 (1.56%)
Mean CpG coverage depth (10×)	44	49	64	60
CpG/total C	22.62%	23.35%	22.13%	21.78%
CpG methylation (mCpG/CpG)	28%	26.6%	27.4%	28.7%
Non-CpG methylation	0.3%	0.2%	0.2%	0.2%


*The data describes, for each sample, the total number of reads and number of alignments with a unique best hit (rows 1–2). Average Conversion rate (row 3) refers to the extent (in percent) of C > T conversion by bisulfite treatment. Rows 4 and 5 provide total number of Cytosine and the number of CpG sites covered by RRBS. Row 6 showed the number of CpG site whose coverage is at least 10. Average read coverage depth (row 7) of CpGs. Rows 8–9 provides the ratio of covered CpGs in total cytosines and the ratio of methylated CpGs in covered CpG sites, there is no statistical significant difference (*p* > 0.5) between any two strains. That non-CpG methylation is underrepresented in RRBS is indicated in row 10.*

A previous study simulated the size of reduced representation genome in the mouse as 1.5 million CpG sites, which is around 1.4 percent of whole genome; this represents 7.0 percent of total genomic CpG sites (Chatterjee et al., 2013). Here, CpG sites covered were calculated as (total CpGs detected)/(average CpG coverage) and thus the number of CpG sites ranged from 76.1 to 104.7 million reads which represents, on average, 10% of total genomic CpG sites (**Table 1**). Of these, the number of CpG sites covered by at least 10 reads (CpG_10_) ranged from 1.33 to 1.58 million (**Table 1**). The mean CpG coverage depth ranged from 44 to 64 across the four samples. Non-CpG cytosines (CpH) were rarely methylated (0.2% compared with 28% methylation for CpG cytosines, **Table 1**). Since our data confirmed that ERRBS enriches for CpGs, non-CpG methylation was not considered further. Thus, our dataset represents a sample of roughly 10% of the CpGs in the genome, albeit non-randomly distributed across the genome. Nevertheless, we consider this as a representative sample for comparative methylome analysis and provide a resource for investigation into changes in DNA methylation patterns in liver disease, tumorigenesis, and regeneration in mice.

### DNA Methylation Pattern is Consistent across Mouse Strains

**FIGURE 1:**
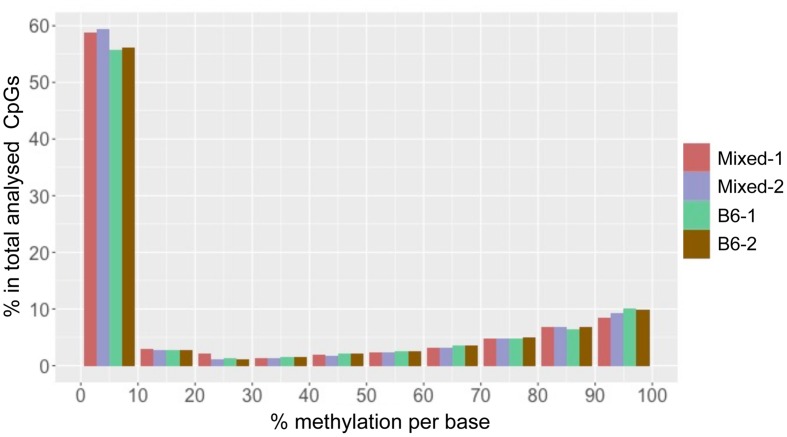
**CpG methylation patterns in mouse liver is consistent across different strains.** The *x*-axis shows percent methylation for each CpG site. The *y*-axis denotes the percentage of CpGs contained in the corresponding bins. Two male mice from either a Mixed or C57BL/6J (B6) are depicted as with red, purple, green and brown, respectively.

We found that total CpG methylation is strikingly similar among the four mouse liver samples from two different genetic backgrounds, ranging from 26.6% to 28.7% (*p* > 0.05 between every two strains; **Table 1**). Analysis of all CpG_10_ sites showed a bimodal distribution of methylation, with nearly 85% of CpG_10_ categorized as either hyper-methylated, defined as >80% of CpGs methylated, or hypo-methylated, defined as <20% methylated. We found remarkable consistency in the methylation patterns, with all samples having 25% of CpG_10_ defined as hypermethylated and 60% as hypomethylated (**Figure 1**). Between 10.4 and 11.4% of the CpG_10_ showed intermediate methylation (>20% and <80% methylated; **Figure 1**).

Reduced representation bisulfite sequencing provides CpG rich regions. In mammalian genomes, it has been shown that CpGis are enriched in annotated gene promoters. Since these CpGis largely unmethylated (Meissner et al., 2008; Illingworth and Bird, 2009), it is expected that the percent methylation found in an RRBS dataset is lower than the average methylation of the whole genome. A methylome study on rat dorsal root ganglia with RRBS, which analyzed 2.8 million CpG sites, demonstrated hypomethylation (0–10% methylated) at more than half of the CpG sites and hypermethylation (90–100% methylated) at about 20% of sites (Hartung et al., 2012). This pattern is similar to what we found in mouse liver. RRBS in mouse embryonic stem cells (mESC) covered 543,678 CpG_10_. These results also displayed a similar methylation pattern to our data in that >40% of CpG sites displayed hypomethylation (Meissner et al., 2008). Therefore, the mouse liver methylome analyzed by ERRBS in our study is similar to RRBS performed on other mouse samples.

**FIGURE 2:**
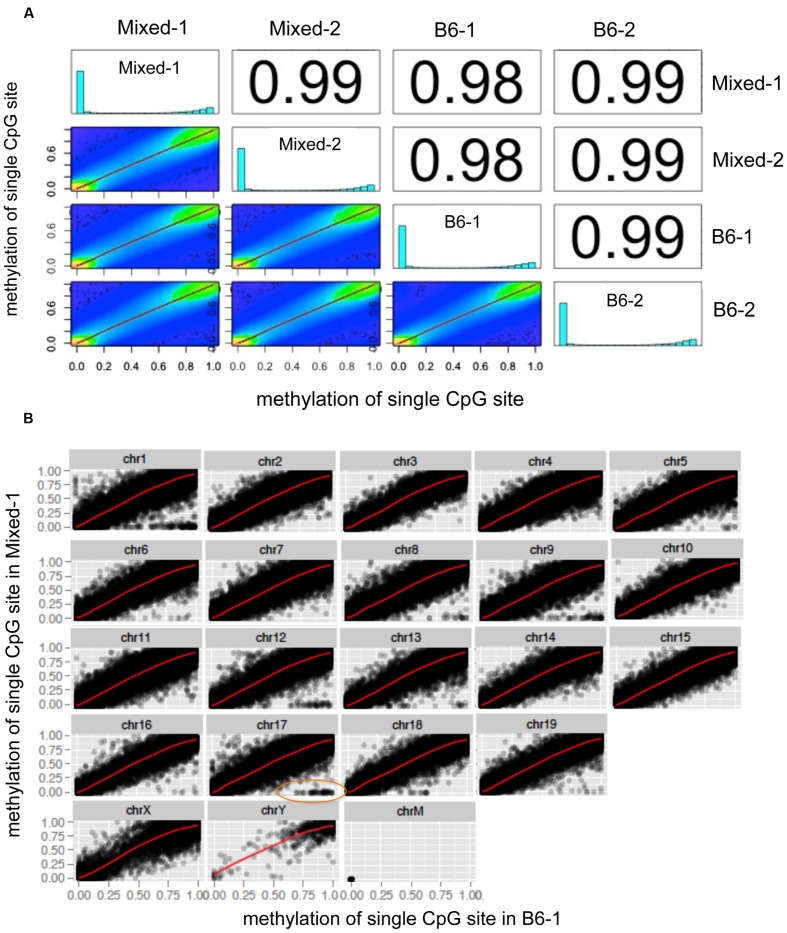
**Global chromosome scatter plots of CpG_10_ sites in mouse.** **(A)** Scatter plot and correlation of CpG methylation across four mice. Numbers on the upper right corner denote pair-wise Pearson’s correlation scores. Histograms on the diagonal denote distribution of methylation patterns of CpG sites for each stain. **(B)** Scatter plots of paired CpGs by chromosome, which was aligned between Mixed-1 and B6-1. Differentiated CpGs in orange ellipse go for further analysis in **Supplementary Figure S1**.

A global chromosome scatter plot of CpG_10_ sites revealed a high correlation between methylation levels at each CpG analyzed across different mouse livers, with Pearson’s correlation coefficient between 0.98 and 0.99 (**Figure 2A**). Subsequently, we generated scatter plots comparing all detected CpG sites on each chromosome between any two samples (**Figure 2B** and **Supplementary Figure S1**). In this analysis, Mixed-1 and B6-1, which represent two samples from different genetic backgrounds, had the lowest Pearson’s coefficient (**Figures 2A,B**). Scatter plots of paired CpGs from these two samples were displayed by chromosome (**Figure 2B**), demonstrating their relationships were highly positively correlated, which is, in part, attributed to the high depth of coverage of these samples (**Table 1**). On the other hand, considering variance brought by different strains, we also did further analysis of the regions that are variable between different strains and found that some CpGs methylation are indeed more correlated within strain than inter strains (**Supplementary Figure S1**). But this kind of variable CpGs only account less than 0.05% of total CpG10, which is too rare to change the global pattern.

Scatter plots with this pattern were also formed between comparisons of all the other samples (**Supplementary Figures S2A,B**). This data demonstrates a remarkable similarity in the methylation profile of male mice, regardless of strain and housing differences.

### The Intra Species CpG Methylation Pattern is Highly Conserved

In general, comparative transcriptome studies have found that gene expression patterns are similar in the same organs across different species and embryos at comparable developmental stages have common gene expression profiles which are, in part, thought to be mediated by methylation patterns in enhancers (Bogdanović et al., 2016), whereas there are dramatic differences in gene expression between different organs from the same species (Zheng-Bradley et al., 2010; Gu et al., 2016). To test whether the same patterns hold true for global CpG methylation, we retrieved and analyzed RRBS data sets from GEO profiling methylomes of zebrafish liver (GSM1456413) the brain from mouse (GSM1069659) and zebrafish (GSE59916; Chatterjee et al., 2014).

**FIGURE 3:**
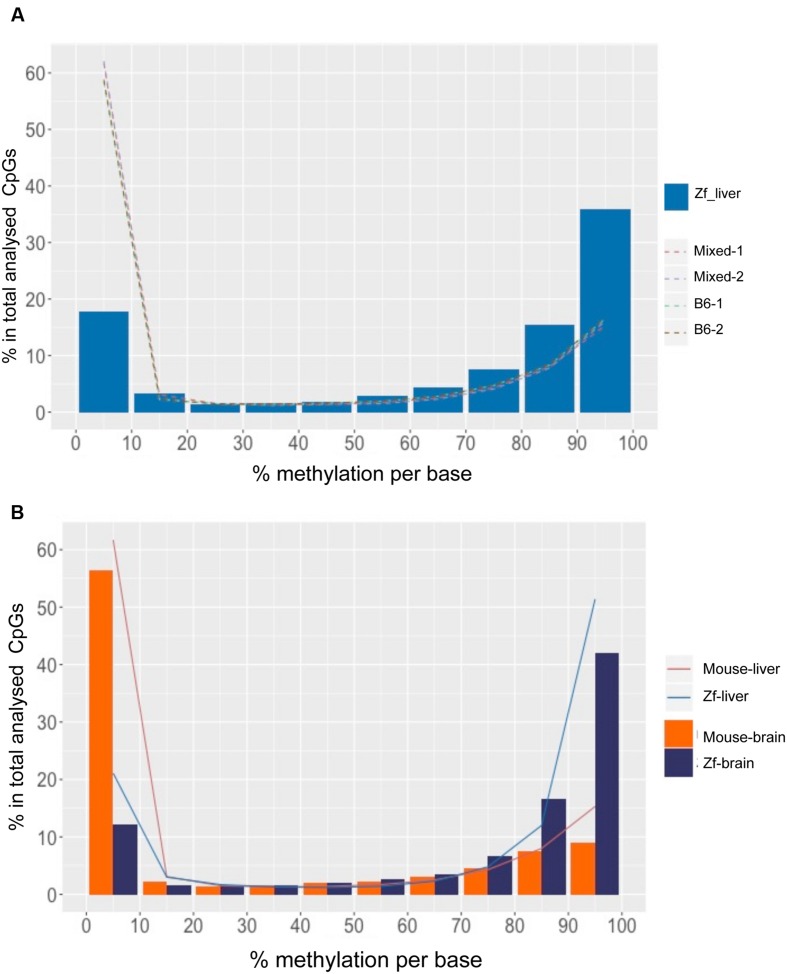
**DNA Methylation patterns are more conserved intra species than the organs within a species.** **(A)** CpG methylation pattern in zebrafish liver is different from mouse liver. The *x*-axis shows percent methylation for each CpG sites. The *y*-axis denotes the percentage of CpGs contained in the corresponding bins. Zebrafish liver is descripted with bars and four mouse strains are shown with dotted lines. **(B)** CpG methylation patterns of intra species are more consistent. The *x*-axis shows percent methylation for each CpG sites. The *y*-axis denotes the percentage of CpGs contained in the corresponding bins. Brain samples are descripted with bars and liver samples are shown with lines.

As in mouse liver and in many other species, the methylome in zebrafish liver displayed a bimodal methylation distribution, with over 60% of CpGs categorized as methylated (>80% methylation) and 15% of CpGs as unmethylated (<20% methylation), however, with an opposite pattern: the zebrafish liver showed much higher prevalence of hypermethylated CpG than mouse liver (*p* < 0.01; **Table 2**), with over 50% and less than 20% of hyper and hypo-methylated CpG sites, respectively (**Figure 3A**, **Table 2**). This is consistent with the finding of between 70 and 85% methylation in whole zebrafish embryos (Potok et al., 2013; Bogdanović et al., 2016) and over 70% methylation in adult muscle (Potok et al., 2013). Thus, the zebrafish liver is more highly methylated than the mouse.

**Table 2:** Methylation levels are more consistent across species than across organs.

	Hypomethylated CpG (<20%)			Hypermethylated CpG > 80%		
		
Organ	Liver	Brain	*p*-value	Liver	Brain	*p*-value
Mouse	62.7%	61.1%	0.5	24.35%	24.2%	0.5805
Zebrafish	24.1%	14.78%	0.03	63.4%	68.9%	0.2692
*p*-value	2.35*e* - 09	2.32*e* - 14		2.35*e* - 09	2.34*e* - 11	


*Ratios are the percent of methylated CpGs out of all the CpG_10_ detected. The *p*-value on the right is between liver and brain in mouse and zebrafish. The *p*-value on the bottom is between mouse and zebrafish in liver and brain.*

To determine whether high levels of methylation in zebrafish could be related to physiological differences between these species, we compared the liver methylomes of both species to that of a very different organ (the brain) within the same species. We found that the same pattern uncovered in the liver was conserved in the brain of both species, with a significantly increased percent of hypermethylated CpGs in zebrafish tissues compared to mouse (*p* < 0.01, **Figure 3B**, **Table 2**). Intraspecies comparison between brain and liver revealed that the mouse brain displayed similar distribution of methylation levels as the mouse liver (*p* > 0.05), with 61.1% of CpG_10_ are hypo-methylated and 24.2% of hyper-methylated (**Table 2**). This same intraspecies conservation was observed in zebrafish, where over 63.4% of the CpG_10_ sites were hyper-methylated and less that 24.1% were hypomethylated in both organs (**Table 2**). Although slightly higher levels of hyper-methylated CpGs were observed in the zebrafish brain (*p* > 0.01), global CpG methylation distributions are still highly consistent between liver and brain of zebrafish (**Figure 3A**). Based on methylome comparisons between different species, different genetic backgrounds, and different organs, we concluded that CpG methylation patterns are more conserved between different organs within a species than between different species for the same organ. Moreover, consistent with findings from other species (Feng et al., 2010; Bogdanović et al., 2016), in both mouse and zebrafish, CpG methylation conforms to a bimodal patterns whereby cytosines are either entirely methylated or unmethylated.

### Hepatic Methylome is Enriched in Intragenic Regions and Introns

**FIGURE 4:**
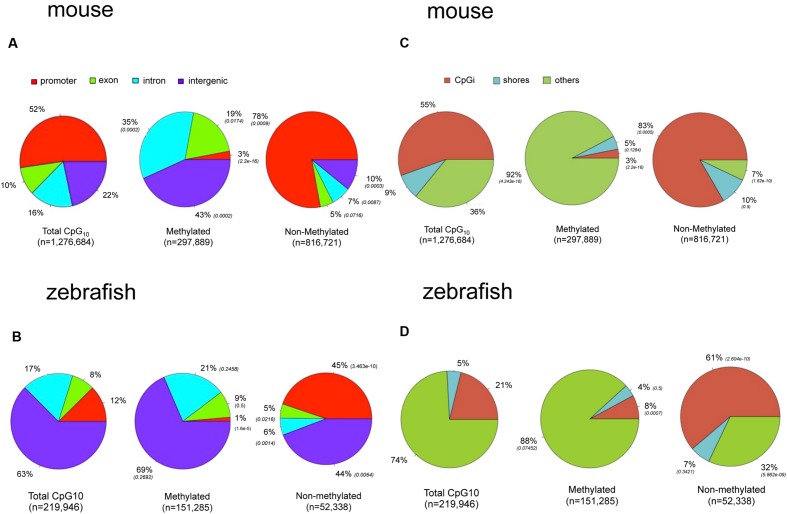
**There are marked differences in the methylome landscapes between the zebrafish and mouse hepatic methylome.** Total CpG_10_ is displayed in the left pie of each panel. Methylated is defined as >80% CpGs methylated and non-methylated is defined as <20% methylated. *P*-value (Chi-square test) in parentheses is to total CpG_10_. **(A)** Gene body for mouse. **(B)** Gene body for zebrafish. **(C)** CpG elements for mouse. **(D)** CpG elements for zebrafish.

To determine if the landscape of methylated CpGs differed between mouse and zebrafish, we compared their distribution relative to the genomic features of hepatic methylomes in both species. All analyzed CpG_10_ sites in both liver datasets were classified into annotated regions. In the mouse, 52% of the CpG_10_ dinucleotides were in promoter regions and 55% were in CpGi (**Figures 4A,C**). This is consistent with the observation that most CpGis are found near sites of transcription initiation (Deaton and Bird, 2011). Other CpG sites are found in exons, introns, and intergenic regions accounting for 10, 16, and 22% of total CpGs levels, respectively (**Figure 4A**). We categorized hypermethylated CpGs as those with >80% of the reads as methylated and hypomethylated CpGs as <20% of reads as unmethylated. Hypermethylated CpGs were intergenic and intronic CpG_10_ (43 and 35%, respectively; *p* < 0.01; **Figure 4A**), and excluded from promoters, and 78% of hypo-methylated CpG_10_ were in annotated promoters (**Figure 4A**). This, in part, is reflective of the distribution of the CpG_10_ in the mouse dataset, of which 52% were mapped to annotated promoters (**Figure 4A**).

**FIGURE 5:**
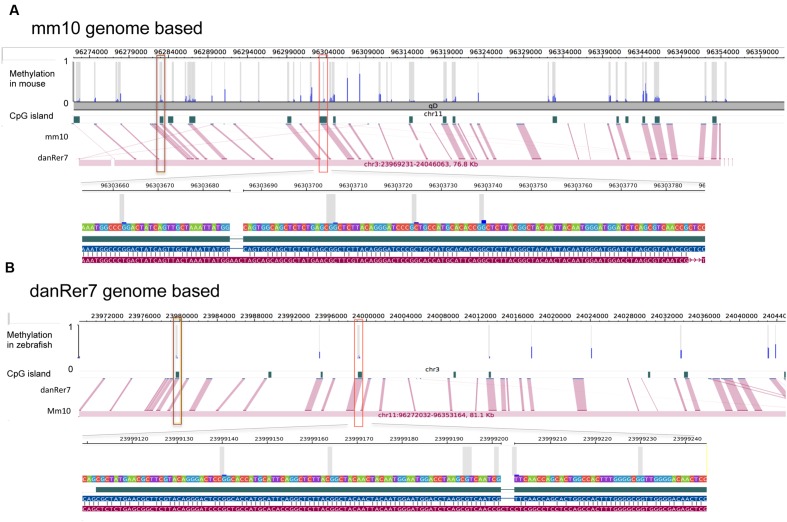
**Methylation of shared CpG islands in mouse and zebrafish genome are overall at a low level.** Each detected CpG site is marked with gray bar and methylation status is shown by blue bar ranging from 0 to 1. CpG islands are annotated based on reference genome mm10 **(A)** and danRer7 **(B)**, respectively. Pinked lines link corresponding CpG islands, two orthologous CpG islands are marked with brown and red squares.

The zebrafish hepatic methylation landscape was strikingly different, with 63% of all CpG_10_ falling within intergenic regions (compared to 22% in mouse) and only 12% within promoter regions (compared to 52% in mouse; **Figure 4B**). The methylated CpG_10_ in zebrafish largely mirrors the genomic distribution of CpGs in this dataset: the majority are found in intragenic regions and introns (69 and 21%, respectively). Promoter regions are the exception to the pattern, as there is a significant exclusion of methylated CpGs from zebrafish promoters (*p* = 1.6 × 10^-6^; **Figure 4B**), similar to the pattern in the mouse hepatic methylome (**Figure 4A**). Other similarities between species include a relative low percent of methylated CpG_10_ in CpGis (21% in zebrafish; 55% in mouse; **Figures 4C,D**) and that the vast majority of methylated CpG_10_ were found in shores and other regions (i.e., open sea), respectively (**Figures 4C,D**). Therefore, although there are striking differences in the methylome landscape between the same tissue in these two species, the finding that CpGis are in general, not methylated (i.e., NMIs), is consistent with findings from other species (Irizarry, 2009; Zemach et al., 2010; Long et al., 2013). We examined several orthologous CpG islands in mouse and zebrafish genome to determine whether the level of methylation was conserved across similar genomic regions. Given that methylation of CpGis is, in general, very low, (**Figures 4C,D**), many orthologous CpGs are unmethylated in the liver of both species. One example is the orthologous CpGi on chromosome 11 in mice and chromosome 7 in zebrafish, which was unmethylated in both species (**Figure 5**).

**FIGURE 6:**
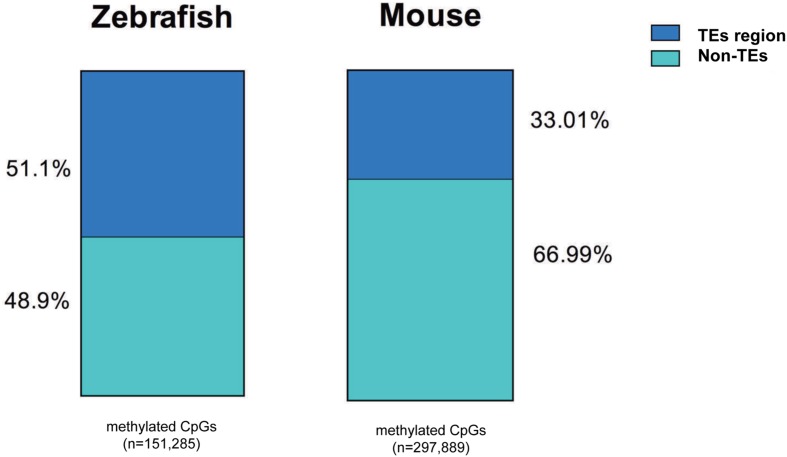
**More methylated CpGs overlap with TEs in zebrafish liver methylome.** All the methylated CpG_10_ covered are annotated with TEs in mouse and zebrafish genome. TEs-methylated CpGs are displayed with blue and non-TEs methylated CpGs are shown with green. The number of CpG sites is under the bar.

We found a much higher percent of CpGs in intragenic regions of the zebrafish genome are methylated in the liver compared to mouse (82% vs. 60%, respectively). This is consistent with the overall higher level of TEs in the zebrafish genome (**Table 3**) and similar to the findings of others (Feng et al., 2010; Potok et al., 2013). To determine whether the high level of methylation in the zebrafish intragenic region was attributed to TE abundance, we examined where methylated CpGs reside in mouse and zebrafish (**Figure 6**). We found that more than half of the methylated CpGs in zebrafish are found in TEs, while in the mouse only one third methylated CpGs are in TEs.

**Table 3:** There is a higher prevalence of repeats in the zebrafish genome compared to the mouse.

	Mouse (Mm10)	Zebrafish (GRCz10)
Genome size	2,652,783,500 bp	1,464,443,456 bp
Total bp in TEs	1,201,953,154 bp (45.3%)	756,790,665 bp (51.8%)
TEs overlap with CpGi	413,896 (0.028%)	32,416 (0.004%)
TEs overlap with CpG10	274,594 (17.63%)	117,032 (53.21%)^∗^


*TEs are the repeat sequence screened by RepeatMasker, the ratio is out of corresponding genome. In zebrafish TEs is significantly higher prevalent that mouse (*p* = 0.0323). CpGi is annotated from UCSC table browser, the ratio in bracket is out of TEs in row 2. CpG10 are those analyzed in our datasets, ration accounts for TEs in row 2. There are significant more TEs overlapping with CpG10 in zebrafish than in mouse (marked with asterisk, *p* < 0.01).*

**FIGURE 7:**
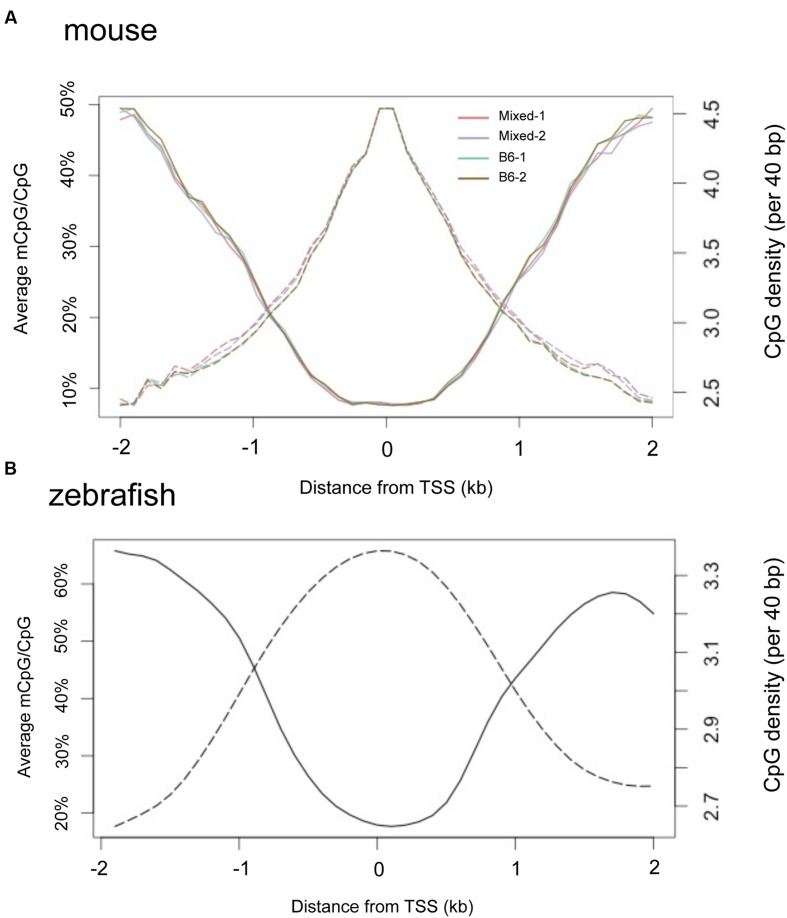
**TSS regions have higher CpG density and less methylation in both mouse and zebrafish livers.** Composite profiles the regions are 2 kb around TSS. *Y*-axis on the left denotes the average of methylated CpG, represented by a solid line. *Y*-axis on the right hand denotes the CpG density, represented by dashed lines. **(A)** Livers from four mice strains. **(B)** Zebrafish liver.

It is widely observed that CpGis cluster in promoters and the regions flanking TSS. We found a negative correlation between methylation level and proximity to the TSS in adult liver methylomes in both mouse (**Figure 7A**) and zebrafish (**Figure 7B**). Notably, the TSS plot revealed a high correlation between the methylation patterns of the four separate mouse liver samples, confirming our finding that there is little variation in the hepatic methylome within mice.

### Correlation between DNA Methylation of Gene Regulatory Regions and Gene Expression

DNA methylation is proposed to repress gene expression. Given that most promoters are not methylated (**Figures 7A,B**), it is difficult to envision how methylation could be a fundamental mechanism that regulates gene expression, except in cases that depart from this pattern and have high methylation levels across their promoters. To examine this further, we counted the number of CpG_10_ within -5 kb upstream of the TSS and ±2 kb TSS regions that were either methylated (>80% of reads methylated) or unmethylated (<20% of reads as methylated), and those with the highest number of methylated CpG_10_ were counted as “hypermethylated” genes and those with the highest number of unmethylated CpG_10_ were counted as “hypomethylated” genes (Supplementary Table 1). Genes with no CpGs were excluded. In both gene lists, we found CpGs are enriched in the TSS regions, but methylation status is different. Examples of genes in both categories are shown in **Supplementary Figure S3**. We noted that there are far fewer CpGs in genes categorized as hypermethylated compared to hypomethylated genes (Supplementary Tables S1 and S2). This indicates that regions with lower CpG content are higher methylated than those with more CpGs, consistent with the finding of others (Lee et al., 2015).

**FIGURE 8:**
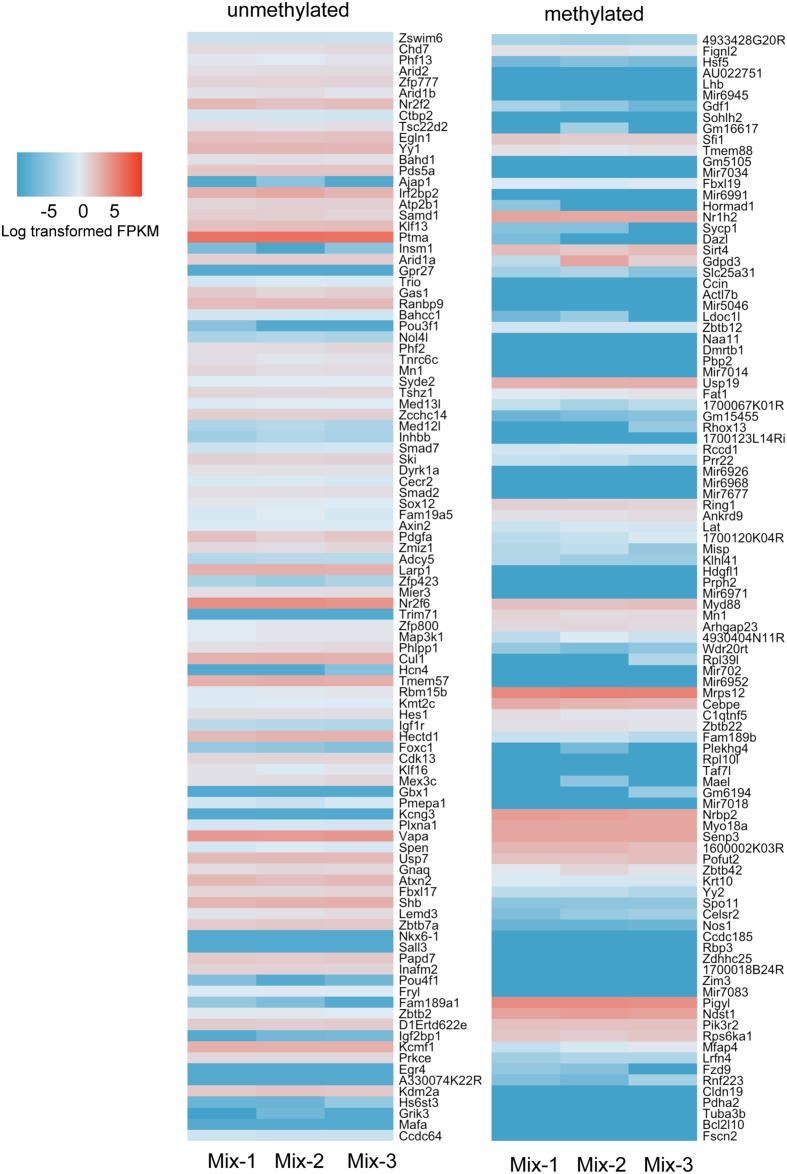
**More hypermethylated genes are expressed at lower levels than hypomethylated genes.** Top 100 genes ranked in hypermethylated (>80%) and hypomethylated (<20%) gene list were plotted with expression values. The gene list was based on the counting CpG in promoter regions (5 kb upstream of TSS). Color scale was represented by the log transformed fpkm value from RNAseq.

We carried out RNAseq analysis on samples from the Mix-1 and Mix-2 livers used for ERRBS as well as of a third sample from a third male mouse from the same background and queried the expression of genes annotated as high and low methylated. We showed the top 100 genes from the methylated and unmethylated gene lists of -5 kb upstream of the TSS in **Figure 8**. To include CpG methylation information in gene bodies, we also count methylated and unmethylated genes based on ±2 kb TSS regions (**Supplementary Figure S4**). Overall, we found in both categories there are more genes in the unmethylated list that are expressed at higher levels than those in the methylated category (**Figure 8** and **Supplementary Figure S4**) However, there are numerous exceptions, as many of the genes categorized as methylated were expressed at high levels, and vise versa, many of the genes which had very low methylation level were expressed also at relative low levels. Together, these data are consistent with findings across a range of species where there is a modest trend in the correlation between promoter methylation and gene expression, but clearly shows that methylation is neither necessary nor sufficient to suppress expression of many genes in the mouse liver.

## Discussion

Reduced representation bisulfite sequencing is a cost effective approach to study global DNA methylation by enriching GC-rich genomic regions. Although a significant number of non-CpG loci can also be detected by RRBS, the overlap between RRBS and whole genome bisulfite sequencing is much greater for CpG loci than non-CpG loci. A study on human embryonic stem cells (hESCs) by whole genome sequencing detected 830,000 CpG sites (Ziller et al., 2011). On the other hand, whole genome bisulfite sequencing across human cell types detected 250,000 non-CpG loci while RRBS detected 213,000 non-CpG loci with an overlap of 52,000 loci (<25%; Ziller et al., 2011). These observations support the use of RRBS as an excellent method for measuring CpG methylation.

This being said, RRBS has limited coverage of intergenic regions and CpGi shores, which are important genomic features. ERRBS is enhanced RRBS, which has been used to resolve DNA methylation patterns in human and other animals (Garrett-Bakelman et al., 2015). It is an optimized RRBS by two ways: (1) biochemically, fragments size selection is optimized to capture more CpG sites by the combination of two enzymes, TaqαI and MspI, and (2) bioinformatically, alignment algorithm is optimized to increase mapping efficiency (Akalin et al., 2012). When compared to RRBS, ERRBS resulted in a higher number of CpGs represented in the data generated and increased coverage of all genomic regions interrogated (Lee et al., 2014). So examining regions of the genome not covered by this method could potentially yield a new perspective on the comparative methylome. A caveat to all bisulfite based methylome approaches is the end is the inability to discriminate between 5mC and 5hmC, however, given that 5hmC represents only a small fraction of all methylated CpGs, this is more of a concern when the direct role of specific CpGs is under investigation. Additional consideration for the ERRBS and RRBS datasets is that the distribution of mapped reads is not entirely random. However, considering the considerable advantage in sequencing depth compared to whole genome bisulfite sequencing, (E)RRBS allows analysis of CpGs methylation in each major genomic element and provides an excellent approach for comparative analysis of the methylome landscape.

The current study is among the first comparisons of the methylome from the same tissue of mouse and zebrafish. A previous study focusing on NMI distribution and function among several vertebrates, including mouse and zebrafish, identified more NMIs that were shared between liver and testis in zebrafish compared to mouse (18854 vs. 16680, respectively), and an expanded number of unique NMIs in the zebrafish liver compared to those that were unique to zebrafish testis (Long et al., 2013). Interestingly, many genes marked as unique NMIs in testis or liver were differentially expressed in these two tissues in mouse, human chicken and platypus samples (Long et al., 2013). Another study comparing the methylation profile of whole zebrafish and mouse embryos found very high levels of CpG methylation in both (74% in zebrafish and 80% in mouse; Feng et al., 2010) and showed a similar pattern of methylated CpGs across gene bodies, repeats and TEs (Feng et al., 2010). The differences between our findings of greatly divergent methylation levels between the mouse and fish hepatic and brain methylomes could be attributed to a difference in tissue studied, as the mouse embryo may have more CpG methylation than a differentiated tissue, or in the method used to interrogate the methylome. Indeed, a comparison between the methylation patterns in the whole early zebrafish embryo to that of adult differentiated muscle revealed that about 70% of CpGs were hypermethylated in muscle, a slightly lower level than observed in pre-gastrula embryos at the sphere and 256 cell stage (>90%; Potok et al., 2013; Bogdanović et al., 2016). Thus, our finding that CpG methylation in zebrafish liver and brain has ranges from 63 to 69% demonstrates that the zebrafish genome during both embryonic development and in differentiated tissues is highly methylated.

A second observation from our work is that the zebrafish genome has a much higher level of CpG methylation compared to the mouse genome. This is probably attributed to an increased prevalence of intergenic regions in zebrafish than that in mouse, as annotated intergenic regions tend to be enriched for methylated CpGs in both mouse and zebrafish (see **Figures 4** and **6** and **Table 3**). This observation is also in line with the presence of more TEs in intergenic regions, in the zebrafish genome than that in the mouse genome (Kapusta et al., 2013). Considering the genome size and repeat sequence proportion individually, TEs comprise 51.8% of the zebrafish genome and 45.3% in the mouse genome (**Table 3**). The finding that the fraction of the genome occupied by TEs is higher in zebrafish can be attributed to the huge amplification of DNA transposons in zebrafish. In fact, the zebrafish is the only vertebrate studied which displays such a dramatic and recent amplification of TEs (Chalopin et al., 2015). It is likely that the zebrafish genome is loaded with potentially active elements, (Lam et al., 1996; Hagemann and Hammer, 2006) which can rapidly amplify their population across the genome and DNA methylation serves as a primary mechanism to silence these. We propose that the high level of DNA methylation in zebrafish is a consequence of this TE-enriched genome and CpG islands, as a mechanism of limiting transposition. Indeed, we find that most of the methylated CpGs in the intragenic region map to the TEs in zebrafish (**Figure 6**). In contrast, the mouse genome is dominated by L1 and SINEs, which are also active but most of the progenitors have accumulated inactivating mutations (Kapusta et al., 2013). Thus, it is very likely that the difference in methylation results from the different evolutionary dynamics of TEs in these two genomes (Chalopin et al., 2015). These findings underscore the concept that the major role for DNA methylation is not only to play prominent role of DNA methylation in regulating gene expression, but also repress expression of repetitive sequences.

## Author Contributions

CZ fulfilled major work of this study, including data analysis, making figure and writing manuscript. During this process, KCS and YH both gave insightful guidance on epigenetic question and bioinformatics methods.

## Conflict of Interest Statement

The authors declare that the research was conducted in the absence of any commercial or financial relationships that could be construed as a potential conflict of interest.

## Footnotes

1 http://www.r-project.org/

2 https://genome.ucsc.edu/

3 http://epigenomegateway.wustl.edu/browser/

## Supplementary Material

**FIGURE S1 | Differentially methylated CpGs between mouse strains are consistent within strains.** Scatter plots of differentiated CpGs, which are marked in **Figure 2**, between Mixed-1 and B6-1 **(A)**, B6-1 and B6-2 **(B)** and Mixed-1 and Mixed-2 **(C)**.

**FIGURE S2 | Global chromosome scatter plots of CpG_10_ sites in mouse livers from different strain combinations.** **(A)** Scatter plots of paired CpGs by chromosome, which was aligned between Mixed-2 and B6-2. **(B)** Scatter plots of paired CpGs by chromosome, which was aligned between B6-1 and B6-2.

**FIGURE S3 | Representative tss region of Hsf5 and Zswim6 displayed different methylation status.** Red bars in the top methylation track showed the status of methylation, whose range is from 0 to 1. The lower reads coverage track displayed TSS regions are enriched and CpG sites were captured by this study were highlighted by red or green. **(A)** Hsf5 represents hyper-methylated genes. **(B)** Zswim6 represents hypo-methylated genes.

**FIGURE S4 | Top 100 genes ranked in hypermethylated (>80%) and hypomethylated (<20%) gene list were plotted with expression values.** The gene list was based on the counting CpG sites in promoter regions and gene bodies (±2 kb of TSS). Color scale was represented by the log transformed fpkm value from RNAseq.
