# Supplementary figures and images for: Comparative Epigenomic Profiling of the DNA Methylome in Mouse and Zebrafish Uncovers High Interspecies Divergence
Source: Front Genet. 2016 Jun 17;7:110. doi: 10.3389/fgene.2016.00110 (PMC4911366; doi:10.3389/fgene.2016.00110)

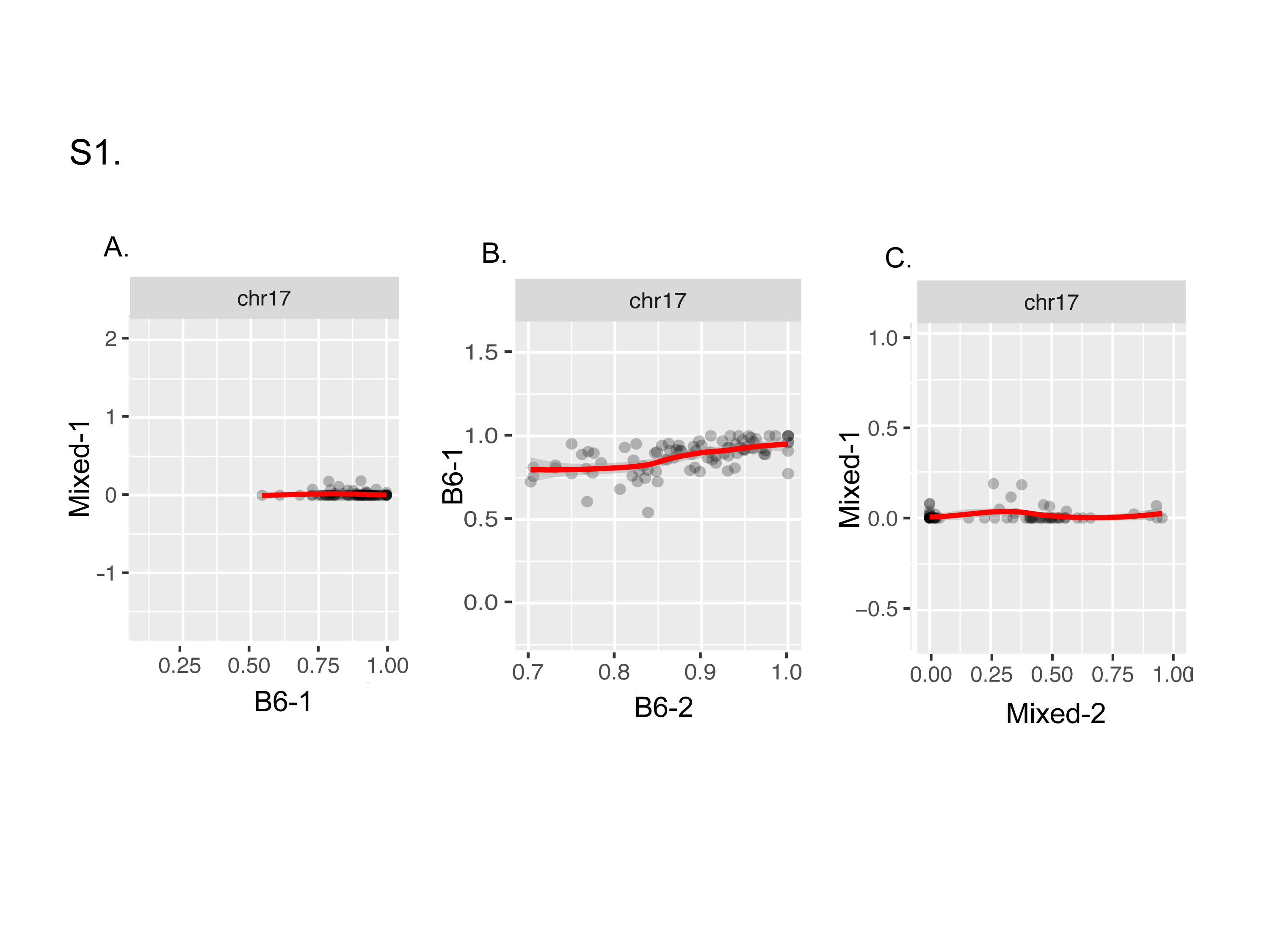

Supplement: Supplementary file 1 [file Image_1.TIF]

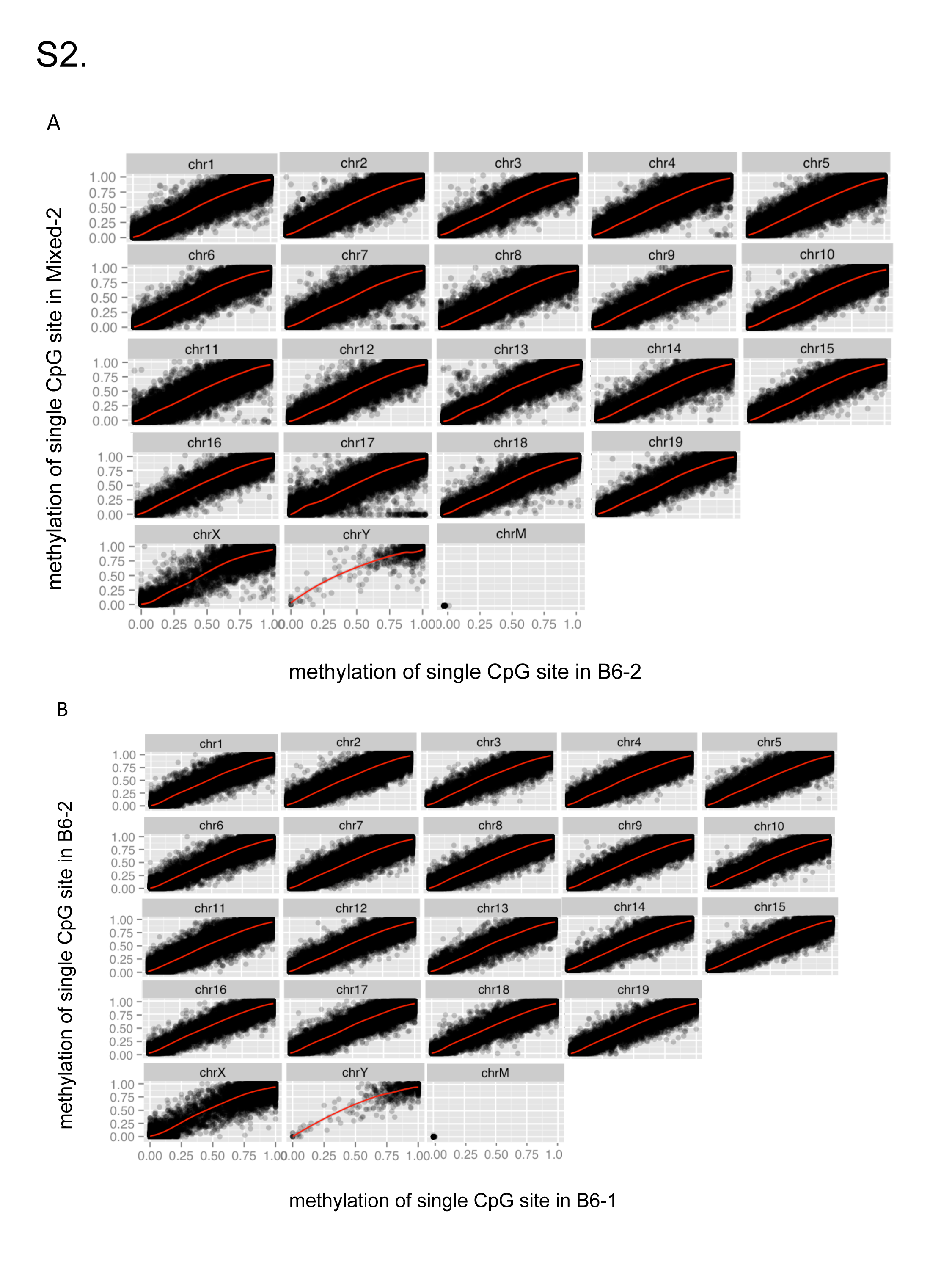

Supplement: Supplementary file 2 [file Image_2.TIF]

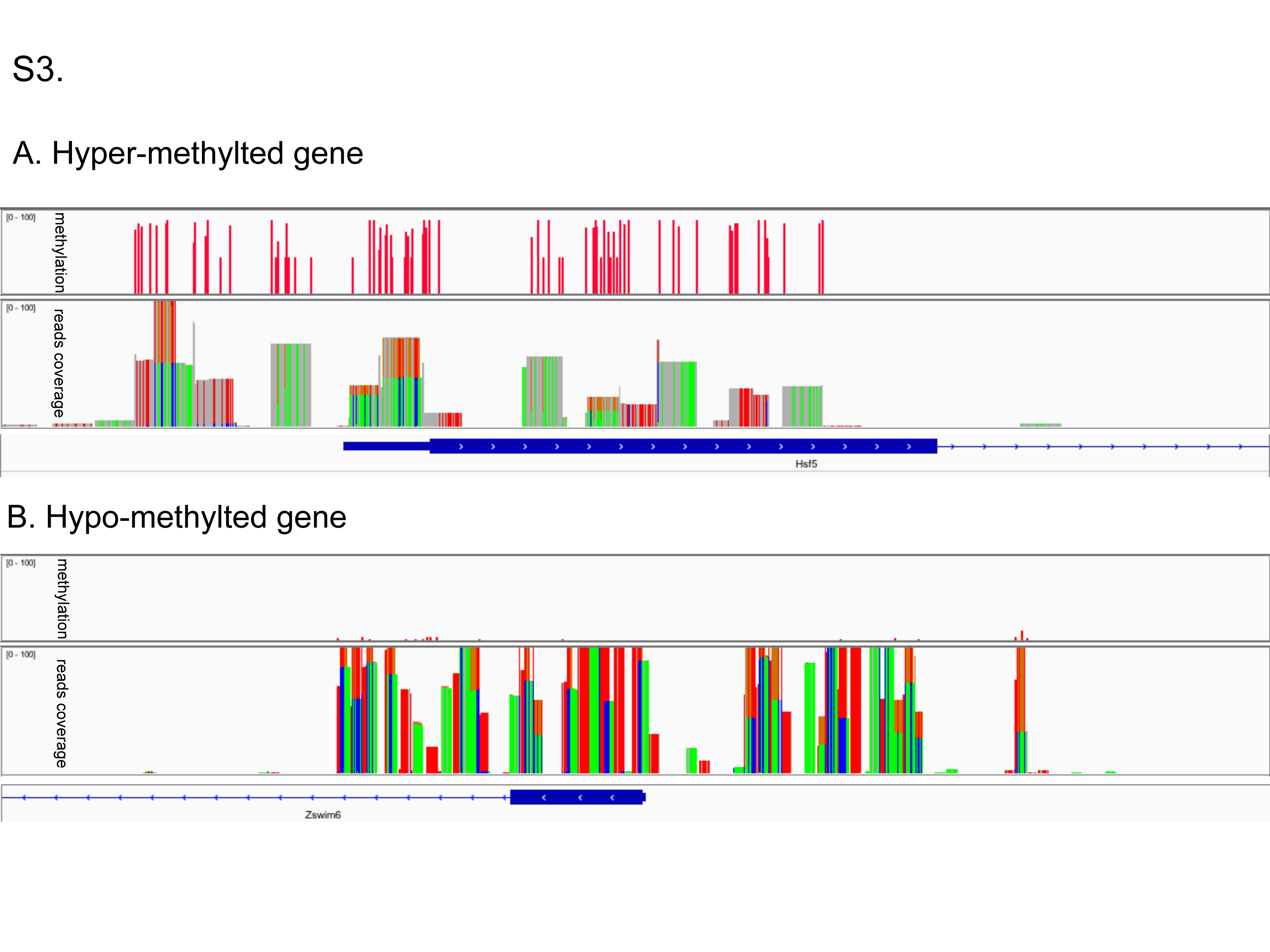

Supplement: Supplementary file 3 [file Image_3.TIF]

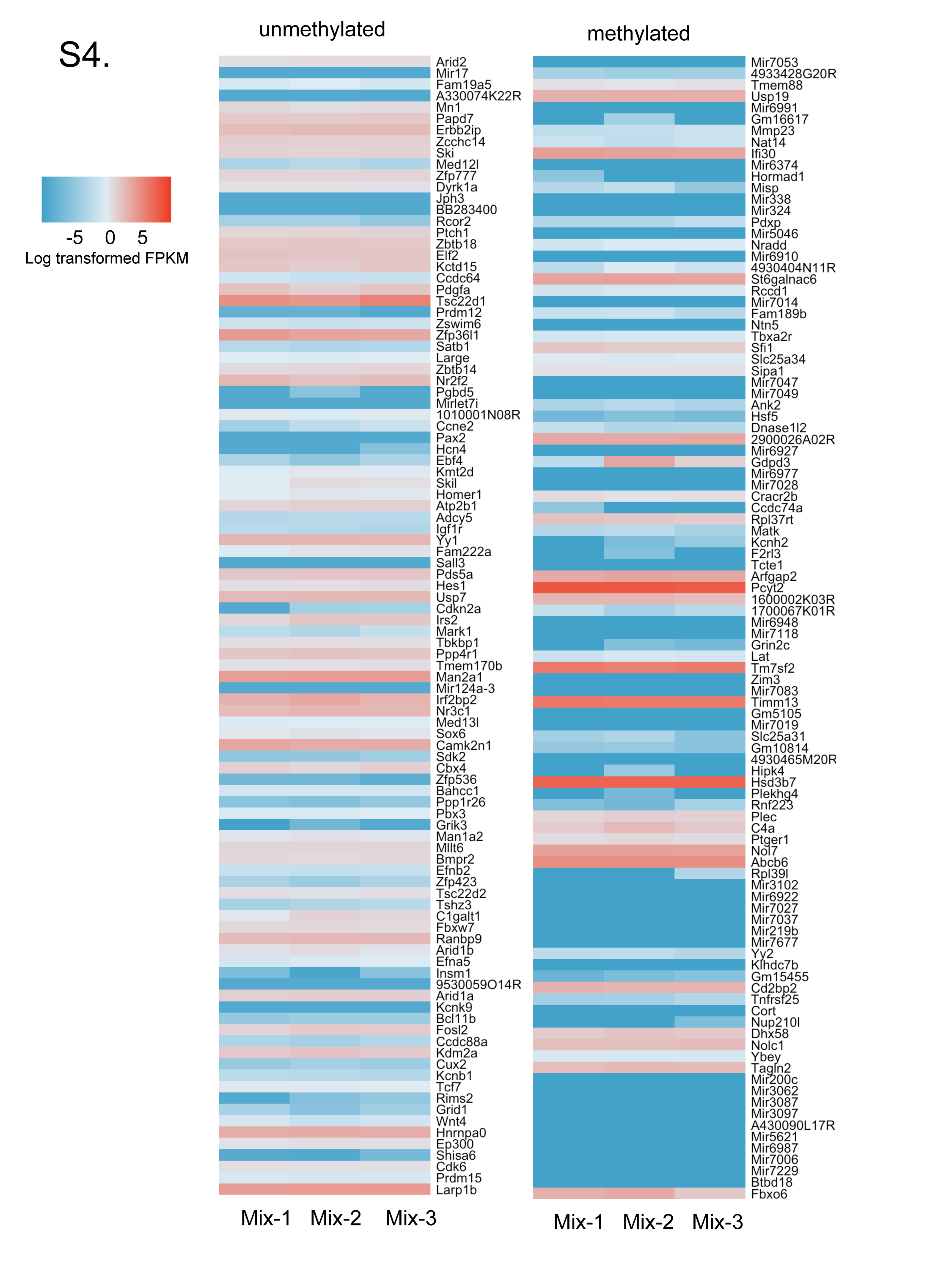

Supplement: Supplementary file 4 [file Image_4.TIF]
