# Supplementary material for: Comparative Epigenomic Profiling of the DNA Methylome in Mouse and Zebrafish Uncovers High Interspecies Divergence
Source: Front Genet. 2016 Jun 17;7:110. doi: 10.3389/fgene.2016.00110 (PMC4911366; doi:10.3389/fgene.2016.00110)
Supplement: Supplementary file 5 [file Data_Sheet_1.DOCX]

**Supp. Table 1. Hyper-methylated gene list**

| **hyper-methylated gene id** | **number of unmethylated CpG** | **number of mCpG** | | **CG content** | **Percent mCpG** |  |
| --- | --- | --- | --- | --- | --- | --- |
| NM_001289118 | 110 | 61 | 171 | | 35.67% | |
| NM_001214911 | 80 | 60 | 140 | | 42.86% | |
| NM_001045527 | 221 | 60 | 281 | | 21.35% | |
| NM_001166433 | 92 | 39 | 131 | | 29.77% | |
| NM_008497 | 59 | 37 | 96 | | 38.54% | |
| NR_105910 | 52 | 36 | 88 | | 40.91% | |
| NR_045728 | 39 | 33 | 72 | | 45.83% | |
| NM_028937 | 57 | 33 | 90 | | 36.67% | |
| NM_008107 | 116 | 33 | 149 | | 22.15% | |
| NM_025915 | 59 | 31 | 90 | | 34.44% | |
| NR_037975 | 100 | 31 | 131 | | 23.66% | |
| NR_106001 | 104 | 31 | 135 | | 22.96% | |
| NM_030207 | 139 | 31 | 170 | | 18.24% | |
| NM_172748 | 152 | 30 | 182 | | 16.48% | |
| NR_105957 | 162 | 30 | 192 | | 15.63% | |
| NM_001289537 | 44 | 29 | 73 | | 39.73% | |
| NM_011516 | 83 | 28 | 111 | | 25.23% | |
| NM_001285517 | 102 | 28 | 130 | | 21.54% | |
| NM_010021 | 142 | 26 | 168 | | 15.48% | |
| NM_001167691 | 59 | 24 | 83 | | 28.92% | |
| NM_178386 | 61 | 24 | 85 | | 28.24% | |
| NM_024228 | 64 | 24 | 88 | | 27.27% | |
| NM_025271 | 92 | 24 | 116 | | 20.69% | |
| NM_001002787 | 94 | 24 | 118 | | 20.34% | |
| NR_039555 | 105 | 24 | 129 | | 18.60% | |
| NM_177630 | 120 | 23 | 143 | | 16.08% | |
| NM_198886 | 34 | 22 | 56 | | 39.29% | |
| NM_019872 | 46 | 22 | 68 | | 32.35% | |
| NR_105981 | 72 | 22 | 94 | | 23.40% | |
| NM_029595 | 95 | 22 | 117 | | 18.80% | |
| NM_001033191 | 161 | 22 | 183 | | 12.02% | |
| NR_003643 | 46 | 21 | 67 | | 31.34% | |
| NM_001185002 | 53 | 21 | 74 | | 28.38% | |
| NM_183097 | 70 | 21 | 91 | | 23.08% | |
| NM_001168372 | 85 | 21 | 106 | | 19.81% | |
| NM_001161816 | 111 | 21 | 132 | | 15.91% | |
| NM_001081286 | 146 | 21 | 167 | | 12.57% | |
| NR_105891 | 48 | 20 | 68 | | 29.41% | |
| NR_105934 | 63 | 20 | 83 | | 24.10% | |
| NR_106142 | 79 | 20 | 99 | | 20.20% | |
| NM_173445 | 90 | 20 | 110 | | 18.18% | |
| NM_001195673 | 93 | 20 | 113 | | 17.70% | |
| NM_175207 | 47 | 19 | 66 | | 28.79% | |
| NM_009066 | 49 | 19 | 68 | | 27.94% | |
| NM_030218 | 50 | 19 | 69 | | 27.54% | |
| NR_027915 | 54 | 19 | 73 | | 26.03% | |
| NR_105937 | 58 | 19 | 77 | | 24.68% | |
| NM_008938 | 80 | 19 | 99 | | 19.19% | |
| NM_010689 | 100 | 19 | 119 | | 15.97% | |
| NM_001081087 | 154 | 19 | 173 | | 10.98% | |
| NM_008232 | 164 | 19 | 183 | | 10.38% | |
| NM_027614 | 30 | 18 | 48 | | 37.50% | |
| NM_010851 | 63 | 18 | 81 | | 22.22% | |
| NR_105917 | 71 | 18 | 89 | | 20.22% | |
| NM_026594 | 75 | 18 | 93 | | 19.35% | |
| NR_030483 | 81 | 18 | 99 | | 18.18% | |
| NM_021493 | 98 | 18 | 116 | | 15.52% | |
| NM_001014836 | 155 | 18 | 173 | | 10.40% | |
| NM_001081235 | 331 | 18 | 349 | | 5.16% | |
| NR_105985 | 64 | 17 | 81 | | 20.99% | |
| NM_028958 | 67 | 17 | 84 | | 20.24% | |
| NM_001081333 | 70 | 17 | 87 | | 19.54% | |
| NR_033512 | 70 | 17 | 87 | | 19.54% | |
| NM_001162933 | 75 | 17 | 92 | | 18.48% | |
| NM_011885 | 76 | 17 | 93 | | 18.28% | |
| NM_207131 | 82 | 17 | 99 | | 17.17% | |
| NM_020625 | 96 | 17 | 113 | | 15.04% | |
| NM_001014995 | 100 | 17 | 117 | | 14.53% | |
| NM_001190319 | 114 | 17 | 131 | | 12.98% | |
| NM_175296 | 151 | 17 | 168 | | 10.12% | |
| NR_003617 | 44 | 16 | 60 | | 26.67% | |
| NR_036631 | 45 | 16 | 61 | | 26.23% | |
| NR_106051 | 47 | 16 | 63 | | 25.40% | |
| NM_008712 | 50 | 16 | 66 | | 24.24% | |
| NM_010660 | 55 | 16 | 71 | | 22.54% | |
| NM_001033547 | 57 | 16 | 73 | | 21.92% | |
| NM_015745 | 59 | 16 | 75 | | 21.33% | |
| NM_144847 | 69 | 16 | 85 | | 18.82% | |
| NM_030702 | 82 | 16 | 98 | | 16.33% | |
| NM_027306 | 89 | 16 | 105 | | 15.24% | |
| NM_030262 | 90 | 16 | 106 | | 15.09% | |
| NM_001098723 | 120 | 16 | 136 | | 11.76% | |
| NM_001100460 | 130 | 16 | 146 | | 10.96% | |
| NM_001083960 | 143 | 16 | 159 | | 10.06% | |
| NM_001004177 | 156 | 16 | 172 | | 9.30% | |
| NM_027207 | 193 | 16 | 209 | | 7.66% | |
| NM_001291212 | 199 | 16 | 215 | | 7.44% | |
| NM_153388 | 41 | 15 | 56 | | 26.79% | |
| NM_010246 | 48 | 15 | 63 | | 23.81% | |
| NM_029568 | 52 | 15 | 67 | | 22.39% | |
| NM_013479 | 56 | 15 | 71 | | 21.13% | |
| NM_001082532 | 59 | 15 | 74 | | 20.27% | |
| NM_001285506 | 63 | 15 | 78 | | 19.23% | |
| NM_008306 | 74 | 15 | 89 | | 16.85% | |
| NM_009449 | 77 | 15 | 92 | | 16.30% | |
| NM_172802 | 81 | 15 | 96 | | 15.63% | |
| NM_001038590 | 136 | 15 | 151 | | 9.93% | |
| NM_001220499 | 158 | 15 | 173 | | 8.67% | |
| NM_008811 | 165 | 15 | 180 | | 8.33% | |
| NM_008841 | 186 | 15 | 201 | | 7.46% | |
| **MAX mCpG** | **MIN mCpG** | **MEDIAN mCpG** | **MAX CG** | | **MIN CG** | |
| 45.83% | 5.16% | 20.21% | 60 | | 15 | |

**Supp. Table 2. Hypo-methylated gene list**

| **hypo-methylated gene id** | **number of unmethylated CpG** | **number of mCpG** | **CG content** | **Percent mCpG** |  |
| --- | --- | --- | --- | --- | --- |
| NM_145456 | 400 | 30 | 430 | 6.98% | |
| NM_001277149 | 340 | 48 | 388 | 12.37% | |
| NM_172705 | 322 | 6 | 328 | 1.83% | |
| NM_175251 | 311 | 50 | 361 | 13.85% | |
| NM_001081382 | 308 | 6 | 314 | 1.91% | |
| NM_001085355 | 305 | 73 | 378 | 19.31% | |
| NM_009697 | 294 | 16 | 310 | 5.16% | |
| NM_009980 | 289 | 60 | 349 | 17.19% | |
| NM_001081229 | 277 | 28 | 305 | 9.18% | |
| NM_053207 | 273 | 10 | 283 | 3.53% | |
| NM_009537 | 271 | 115 | 386 | 29.79% | |
| NM_001045523 | 270 | 24 | 294 | 8.16% | |
| NM_001081321 | 266 | 21 | 287 | 7.32% | |
| NM_001099299 | 263 | 9 | 272 | 3.31% | |
| NM_026482 | 261 | 106 | 367 | 28.88% | |
| NM_001164598 | 261 | 50 | 311 | 16.08% | |
| NM_001081415 | 259 | 104 | 363 | 28.65% | |
| NM_021366 | 254 | 70 | 324 | 21.60% | |
| NM_008972 | 252 | 121 | 373 | 32.44% | |
| NM_016889 | 252 | 121 | 373 | 32.44% | |
| NM_008158 | 251 | 78 | 329 | 23.71% | |
| NM_001080819 | 251 | 22 | 273 | 8.06% | |
| NM_001081302 | 250 | 42 | 292 | 14.38% | |
| NM_019930 | 249 | 61 | 310 | 19.68% | |
| NM_198423 | 249 | 12 | 261 | 4.60% | |
| NM_008086 | 249 | 5 | 254 | 1.97% | |
| NM_011141 | 247 | 101 | 348 | 29.02% | |
| NM_001134300 | 245 | 67 | 312 | 21.47% | |
| NM_011078 | 245 | 58 | 303 | 19.14% | |
| NM_198022 | 245 | 4 | 249 | 1.61% | |
| NM_001166064 | 244 | 105 | 349 | 30.09% | |
| NM_001081235 | 244 | 17 | 261 | 6.51% | |
| NM_172424 | 243 | 109 | 352 | 30.97% | |
| NM_001081300 | 243 | 60 | 303 | 19.80% | |
| NM_080855 | 242 | 73 | 315 | 23.17% | |
| NM_177855 | 241 | 21 | 262 | 8.02% | |
| NM_008381 | 239 | 64 | 303 | 21.12% | |
| NM_011385 | 238 | 74 | 312 | 23.72% | |
| NM_001042660 | 238 | 48 | 286 | 16.78% | |
| NM_007890 | 237 | 95 | 332 | 28.61% | |
| NM_011438 | 236 | 79 | 315 | 25.08% | |
| NM_010754 | 236 | 39 | 275 | 14.18% | |
| NM_001128151 | 236 | 33 | 269 | 12.27% | |
| NM_134096 | 236 | 4 | 240 | 1.67% | |
| NM_015732 | 235 | 9 | 244 | 3.69% | |
| NM_008808 | 234 | 94 | 328 | 28.66% | |
| NM_183208 | 233 | 75 | 308 | 24.35% | |
| NM_001012765 | 232 | 82 | 314 | 26.11% | |
| NM_028451 | 232 | 81 | 313 | 25.88% | |
| NM_172593 | 232 | 39 | 271 | 14.39% | |
| NM_033327 | 232 | 27 | 259 | 10.42% | |
| NM_010150 | 231 | 59 | 290 | 20.34% | |
| NM_001042503 | 230 | 87 | 317 | 27.44% | |
| NM_011945 | 230 | 71 | 301 | 23.59% | |
| NM_133821 | 230 | 49 | 279 | 17.56% | |
| NM_001081678 | 230 | 26 | 256 | 10.16% | |
| NM_012042 | 229 | 68 | 297 | 22.90% | |
| NM_019427 | 228 | 28 | 256 | 10.94% | |
| NM_175402 | 227 | 129 | 356 | 36.24% | |
| NM_001081192 | 227 | 59 | 286 | 20.63% | |
| NM_025382 | 227 | 34 | 261 | 13.03% | |
| NM_001081383 | 226 | 103 | 329 | 31.31% | |
| NM_010513 | 226 | 61 | 287 | 21.25% | |
| NM_008235 | 226 | 60 | 286 | 20.98% | |
| NM_144788 | 225 | 72 | 297 | 24.24% | |
| NM_008592 | 224 | 111 | 335 | 33.13% | |
| NM_027118 | 224 | 34 | 258 | 13.18% | |
| NM_078477 | 223 | 73 | 296 | 24.66% | |
| NM_001039214 | 222 | 31 | 253 | 12.25% | |
| NM_022995 | 221 | 27 | 248 | 10.89% | |
| NM_153512 | 221 | 11 | 232 | 4.74% | |
| NM_015739 | 221 | 7 | 228 | 3.07% | |
| NM_008881 | 220 | 43 | 263 | 16.35% | |
| NM_019763 | 220 | 32 | 252 | 12.70% | |
| NM_013933 | 220 | 28 | 248 | 11.29% | |
| NM_009125 | 219 | 63 | 282 | 22.34% | |
| NM_001003918 | 219 | 53 | 272 | 19.49% | |
| NM_015794 | 219 | 53 | 272 | 19.49% | |
| NM_008139 | 219 | 52 | 271 | 19.19% | |
| NM_001033306 | 218 | 44 | 262 | 16.79% | |
| NM_010731 | 217 | 127 | 344 | 36.92% | |
| NM_001081193 | 217 | 85 | 302 | 28.15% | |
| NM_178280 | 216 | 109 | 325 | 33.54% | |
| NM_144955 | 216 | 56 | 272 | 20.59% | |
| NM_028194 | 215 | 118 | 333 | 35.44% | |
| NM_183087 | 215 | 78 | 293 | 26.62% | |
| NM_001301269 | 215 | 18 | 233 | 7.73% | |
| NM_001169131 | 215 | 7 | 222 | 3.15% | |
| NM_011143 | 215 | 6 | 221 | 2.71% | |
| NM_001033466 | 214 | 14 | 228 | 6.14% | |
| NM_133825 | 213 | 81 | 294 | 27.55% | |
| NM_009951 | 212 | 78 | 290 | 26.90% | |
| NM_019715 | 212 | 55 | 267 | 20.60% | |
| NR_110496 | 211 | 63 | 274 | 22.99% | |
| NM_011104 | 211 | 50 | 261 | 19.16% | |
| NM_020596 | 211 | 50 | 261 | 19.16% | |
| NM_015820 | 209 | 94 | 303 | 31.02% | |
| NM_001001984 | 209 | 65 | 274 | 23.72% | |
| NM_194350 | 208 | 91 | 299 | 30.43% | |
| NM_001081097 | 208 | 53 | 261 | 20.31% | |
| **MAX mCpG** | **MIN mCpG** | **MEDIAN mCpG** | **MAX CGs** | **MIN CGs** | |
| 36.92% | 1.16% | 19.49% | 430 | 221 | |
